# Boron Nitride-Filled Linear Low-Density Polyethylene for Enhanced Thermal Transport: Continuous Extrusion of Micro-Textured Films

**DOI:** 10.3390/polym13193393

**Published:** 2021-10-02

**Authors:** Özgün Güzdemir, Sagar Kanhere, Victor Bermudez, Amod A. Ogale

**Affiliations:** 1 Department of Food Engineering, Adnan Menderes University, Aydın 09010, Turkey; oozdemi@g.clemson.edu; 2 Department of Chemical Engineering, Center for Advanced Engineering Fibers and Films (CAEFF), Clemson University, Clemson, SC 29634, USA; skanher@g.clemson.edu (S.K.); vbermud@g.clemson.edu (V.B.)

**Keywords:** boron nitride, linear low-density polyethylene, micro-textured films, continuous extrusion, thermal conductivity

## Abstract

With shrinking size of electronic devices, increasing performance and accompanying heat dissipation, there is a need for efficient removal of this heat through packaging materials. Polymer materials are attractive packaging materials given their low density and electrical insulating properties, but they lack sufficient thermal conductivity that inhibits heat transfer rate. Hexagonal boron nitride (BN) possesses excellent thermal conductivity and is also electrically insulating, therefore BN-filled polymer composites were investigated in this study. Results showed successful continuous extrusion of BN-filled linear low-density polyethylene through micro-textured dies that is a scalable manufacturing process. Through-thickness thermal conductivity measurements established that 30 vol% BN content led to an over 500% increase in thermal conductivity over that of pure polymer. Textured film surface provided about a 50% increase in surface area when compared with non-textured films. This combination of increased surface area and enhanced thermal conductivity of BN-filled textured films indicates their potential application for improved convective thermal transport.

## 1. Introduction

In the last few decades, the fast rate of development of consumer electronics has resulted in a wide variety of miniaturized devices that run faster processors and are expected to operate for longer periods of time [1]. The increase in performance and usage time lead to a larger heat generation by those devices, which creates discomfort for the user, impairs the device performance, and shortens the life of electronic components, eventually leading to their failure [2,3,4,5]. Therefore, there is a need for enhanced thermal management materials in electronic packaging that are lightweight, easily processable, and cost-efficient [6]. Electronic packaging typically has a large surface area to enable increase in convective heat flow, but large surface of electrically conducting material can also lead to undesired displacement current flow [7].

In general, polymers are an attractive material for electronic packaging because of their light weight, low cost, ease of processing, and electrical insulating properties that prevents noise or voltage drop in the electric signals [6,8,9,10]. However, their use in applications requiring thermal management is limited by their low thermal conductivity, about 0.1–0.5 W/m·K [11]. To overcome this obstacle, highly thermally conductive fillers are incorporated into a polymer matrix to enhance its thermal conductivity [12,13]. Among the most common fillers are metallic particles such as Al, Ag, Cu and Ni powders. Additionally, carbon-based fillers such as graphite, carbon fibers and carbon nanotubes, and ceramic particles such as Al_2_O_3_, SiC, and hexagonal boron nitride (BN) are also used [14,15].

Metal fillers are not desired due to their high density and high electrical conductivity. Carbon-based fillers make light-weight composites but are electrically conducting [6]. In contrast, ceramic fillers enhance polymer thermal conductivity without significantly changing electrically insulating characteristics of the polymer composite samples obtained by compression molding [16].

Among various types of ceramic fillers for polymers being currently explored, BN is receiving particular attention because it is an electrically insulating counterpart of graphite with a wide band gap of 5 eV and low dielectric constant of 3.9 [12]. Several previous studies have investigated the filling of polymer matrices with BN particles [12,13,14,16,17,18,19,20,21,22,23,24]. These studies report that the crystalline structure of boron nitride decreases phonon scattering events that lead to increase in thermal conductivity [25,26]. Electrical insulating characteristics do not change significantly upon adding BN particles to linear low-density polyethylene (LLDPE) [20]. Nevertheless, enhancement of convective heat transport can also be accomplished by increasing the area of the material that is exposed to the cooling media, i.e., to create extended surfaces or fins. For devices such as heat exchanger tubes, the addition of fins provides rigidity that is beneficial to its structural integrity. Potential use of thermally conductive polymer matrices include applications wherein the polymers must be made into a film or fabric, e.g., spacesuits components for removal of an astronaut’s body heat [21], components of full-body suits worn for medical PPE, and flexible displays for foldable electronic devices [25,26]. If flexible films are needed, their bending stiffness must be kept small, for which the extended surfaces must be made very thin and numerous to generate the same total surface area as thicker, fewer fins. This can be achieved by micro-texturing of the film.

However, much of the research on micro-texturing of polymers has focused on applications for tribology, superhydrophobic surfaces, and tissue culturing, among others [27,28,29,30]. Some of the techniques investigated for micro-texturing of polymers are mold casting [27], photolithography [28,29], hot embossing [30], chemical etching [31], and solution spraying [32]. All these methods are batch processes that are too slow and expensive for large-scale production of textured films. In an earlier study [33], we have successfully demonstrated the processing of neat (unfilled) polypropylene into textured films.

In this study, we investigate the continuous melt extrusion of micro-textured BN-filled linear low-density polyethylene composite films because it has not been systematically investigated in the literature. The specific objectives of this study were to: (i) investigate the rheology and the continuous extrusion of BN-filled polyethylene through a micro-textured die, and (ii) evaluate the enhanced heat transport properties of the films as a function of the resulting internal microstructure and surface micro-texture.

## 2. Materials and Methods

### 2.1. Materials

The polymer matrix used throughout this study was poly(ethylene-co-1-octene) (Aspun 6835, Dow Inc., Midland, MI, USA), a flexible linear low-density polyethylene (LLDPE). It has a density of 0.950 g/cm^3^, DSC melting point of 129 °C, and melt flow index of 17 g/10 min (190 °C/2.16 kg, ASTM D1238). LLDPE was chosen because of its light weight, ease of processability, low cost, and flexibility. Hexagonal boron nitride (h-BN) micro-platelets (PT100, Momentive Performance Materials Inc., Waterford, NY, USA) with a mean particle size of 13 µm were used as fillers with a density 2.25 g/cm^3^. LLDPE and BN were supplied in the form of pellets and powder, respectively.

### 2.2. Processing

BN/LLDPE composite films were prepared by melt-extrusion using a 15 mL co-rotating twin-screw MC15HT micro-extruder (DSM Xplore, Geleen, Netherlands). The extruder consisted of two conical screws nominally 160 mm long and having a diameter of 22 mm at the entrance that tapered down to 8 mm at the end. For the purpose of this study, melt-compounding of LLDPE pellets and filler powder was done in a co-rotating mode at 60 rpm, with a barrel temperature of 210 °C. Preliminary studies had revealed that 5 min of mixing time was inadequate, whereas 10 min was found to be adequate to disperse BN within the matrix.

**Figure 1:**
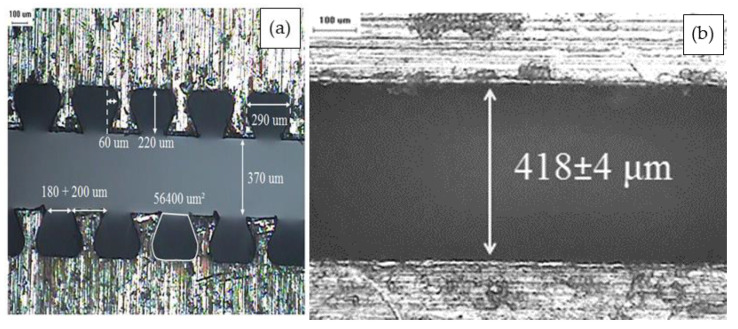
Cross-section of dies used for film extrusion: (**a**) trapezoidal-shaped pattern, and (**b**) non-textured rectangular die.

Textured films were then melt-extruded through a die with trapezoid-shaped cavities that were 220 ± 9 µm high, as shown in Figure 1a Control samples, i.e., non-textured films, were melt-extruded from a flat die with a width of ~420 µm, as shown in Figure 1b. Both textured and non-textured samples with a BN content of 0, 1, 5, 10, 20, and 30 vol% (LLDPE, BN1, BN5, BN10, BN20, and BN30) were prepared.

### 2.3. Characterization

Rheological characterization was performed to investigate the effect of BN reinforcement on film processing. Samples of the BN/LLDPE blends were obtained from the micro-extruder after melt-compounding and rheological tests were performed on an ARES rheometer (TA Instruments, New Castle, DE, USA) with a cone-and-plate fixture of 25 mm in diameter and cone angle of 0.1 rad. Viscosities at high shear rates (100–10,000 s^−1^) and die swell were measured on a capillary rheometer (ACER 2000, Rheometric Scientific, Piscataway, NJ, USA) using a 1-mm diameter capillary die and a 20 MPa transducer. Bagley correction was applied to the capillary rheometer measurements. Viscosity values were fitted to a Cross model shown in Equation (1), where η (γ) is the shear viscosity, γ is the shear rate, λ is the time constant, $\eta_{0}$ is the zero shear viscosity, and *n* is the power law index.

(1) $$\frac{\eta \;(\gamma )}{\eta_{0}}=1/[1+{\left(\lambda \gamma \right)}^{\left(1-n\right)}]$$

For die swell measurements, video images were recorded. A brass spatula was used to rapidly clean the melt from the die exit to prevent the extrudate elongation due to draw-down by gravity. Still frames were extracted from the recording to measure extrudate diameter relative to the circular die using ImageJ software. It is noted that capillary die diameter was 1 mm and outer die block diameter was 22.5 mm (used as reference). Die swell ratio was then calculated using the following standard equation [34].

(2) $$Die\;swell\;ratio=\frac{D_{extrudate}}{D_{die}}-\;0.13$$

Optical microscopy (Olympus BX60 Optical Co., Tokyo, Japan) was used to evaluate the lateral surface area of the BN/LLDPE films. Scanning electron microscopy (SEM, Hitachi S-4800, Hitachi, Japan) was performed to examine the microstructure of the BN/LLDPE films, orientation, and effective dispersion of the BN micro-platelets in the LLDPE matrix.

A laser flash analysis (LFA) technique (LFA447 Nanoflash, Netzsch, Germany) was used to measure the through-thickness thermal conductivity of the BN/LLDPE films by following ASTM E1461-13 standard test method. Square specimens of 10 × 10 × 0.2 mm were prepared for through-thickness measurements. Three replicate specimens were analyzed per composite composition and film type, and three repeated measurements were carried out per specimen. Through-thickness thermal diffusivity values were calculated using Cowan + pulse correction model that accounts for possible heat losses during measurement. Thermal diffusivity values for micro-textured films were calculated using equivalent thickness calculated by dividing cross-sectional area by width of the film from cross-sectional images of the films. 

Tensile properties were measured by tensile testing (ASTM D638-14) using an ATS Universal 900 machine, operating at a crosshead speed of 50 mm/min. Dog-bone-shaped specimens with a length of 25 mm and a width of 5 mm were cut from the BN/LLDPE films for testing in the machine direction (MD). Because the film specimens were produced using a small, lab-scale extruder/die, the width of the film was limited, and so transverse properties could not be measured with adequate accuracy.

## 3. Results and Discussion

### 3.1. Rheology and Processing

#### 3.1.1. Viscosity and Die Swell

**Figure 2:**
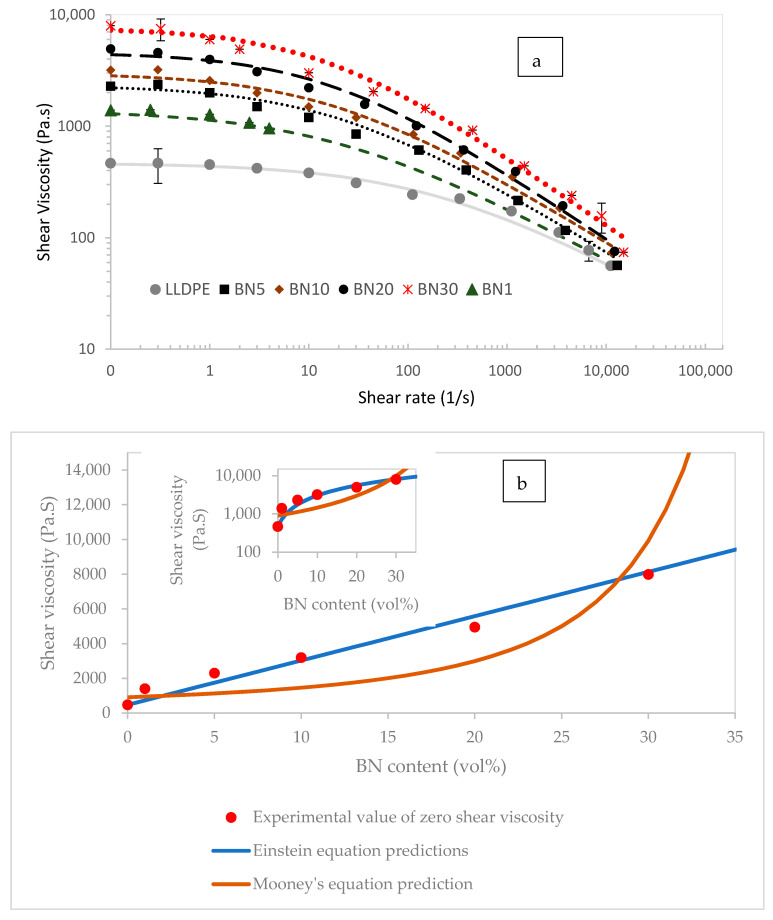
(**a**) Shear viscosity versus shear rate results of neat LLDPE and BN/LLDPE blends at 210 °C. Low shear experiments were performed in a cone-and-plate rheometer, whereas high shear measurements were done in a capillary rheometer. Symbols represent experimental data whereas dotted lines are best fits to Cross model. (**b**) Zero shear viscosity as a function of BN content (data points) and predictions from Mooney and Einstein equations with k_E_ set to 55. Shear viscosity is plotted on linear scale in main plot to emphasize predicted viscosity rise by Mooney’s equation and on log-scale as inset.

Figure 2 displays shear viscosity of the BN/LLDPE blends over a large range of shear rates measured using the two different methods, cone-plate (low shear) and capillary (high shear). As expected, addition of BN powder (solids) resulted in an increase of viscosity with increasing with BN content. Pure LLDPE and BN/LLDPE blends both behaved as pseudo-plastic fluids. However, the Newtonian plateau was wider for pure LLDPE as compared with that for BN/LLDPE blends. Addition of BN to the polymer resulted in more shear thinning behavior, and consequently lower power-law indices, as shown in Table 1. This is consistent with the increase in shear thinning behavior by addition of solids into polymer melts reported in previous literature studies [35].

The viscosity increase with increasing BN content was noted especially at lower shear rates. A significant increase (about an order of magnitude) was observed at 5 vol% BN, but the increase continues for higher BN contents. This concentration (5 vol% or less) might be an indicator of a nominal network of particles that competes with molecular mobility of the polymer melt [36]. Zero shear viscosity of the BN/LLDPE composites is plotted in Figure 2b as a function of BN content together with predictions from Einstein and Mooney equations for suspensions [37,38]. Predictions of Einstein’s equation match well with experimental values at lower-moderate BN contents, but prediction using Mooney’s equation capture the significant rise in viscosity for higher BN contents indicating potential problems during extrusion, as will be discussed later.

**Figure 3:**
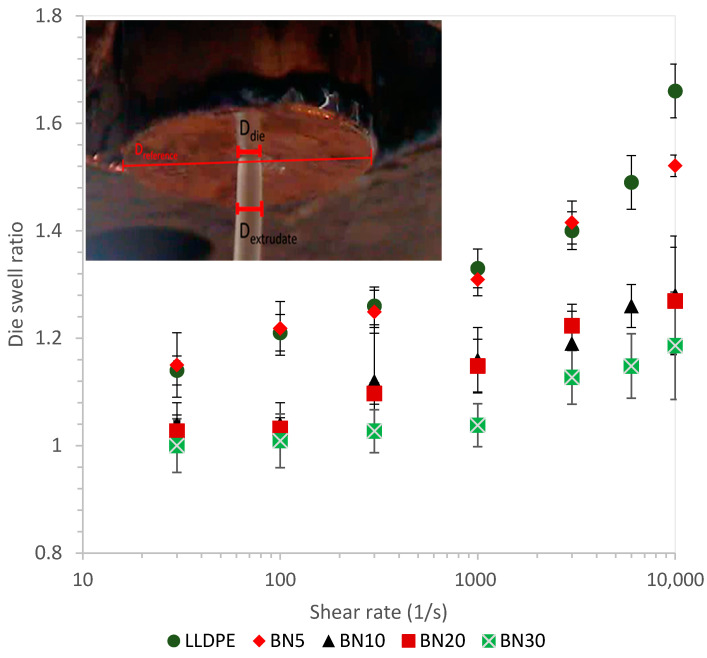
Experimentally measured die swell ratios for LLDPE and BN/LLDPE blends over a range of shear rates. The inset shows a representative still-frame image of the extrudate with the die block, die capillary, and extrudate diameters identified on the picture where capillary D_die_ = 1.0 mm and D_reference_ = 22.5 mm.

When a viscoelastic fluid flows out of a die without any drawing force, the cross-section dimensions of the extrudate are larger than that of the die [39,40]. This is due to the relaxation of primary normal stresses after the melt exits the die wall and there is essentially a free-surface [41,42]. Investigation of such flow behavior is important for film extrusion because for non-circular die shapes, size and shape of the extrudates after extrusion can vary based on extent of die swell [43]. Therefore, die swell ratios for neat LLDPE and BN/LLDPE blends were measured by using the extrudate diameters exiting the capillary rheometer. As displayed in Figure 3, larger die swell ratios were observed at higher shear rates because more elastic energy is stored. For neat LLDPE, the ratio increased from 1.14 ± 0.03 to 1.66 ± 0.05 by increasing the rate from 30 s^−1^ to 10,000 s^−1^, respectively. 

Interestingly, the die swell ratios of the melt extrudate decreased with increasing BN content (for a given shear rate). LLDPE films filled with 30% BN showed die swell ratio of 1.00 ± 0.05 and 1.19 ± 0.10 at the shear rates of 30 s^−1^ to 10,000 s^−1^, respectively. This observation is consistent with previous ones reported in the literature and can be explained by the fact that the elastic recovery is harder for polymer chains with higher filler content than for the corresponding neat polymer chains (that are not constrained by the BN particles). Thus, overall melt elasticity is affected by shear rate and filler content [39].

**Figure 4:**
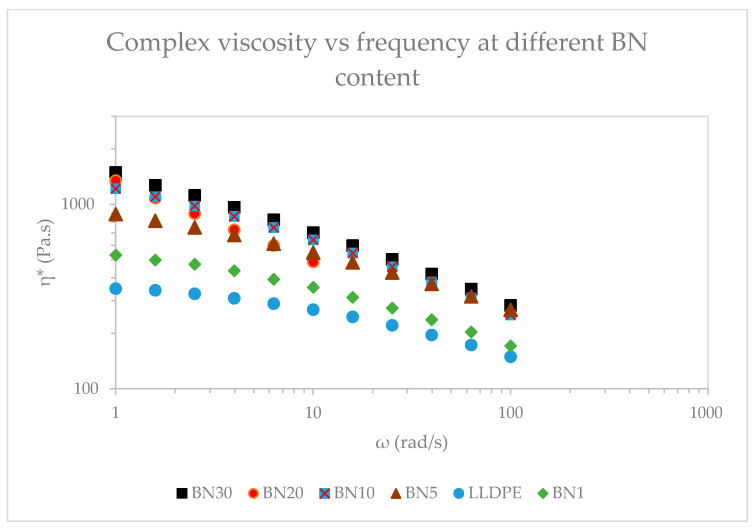
Complex viscosity of BN/LLDPE melts at 210 °C for different BN contents.

Viscoelastic response of BN/LLDPE blend was further investigated by measuring dynamic viscosity at the typical film extrusion temperature of 210 °C. As shown in Figure 4, the complex viscosity of the nanocomposite containing 30 vol% BN is about half an order of magnitude higher than that of pure LLDPE at 1 rad/s, whereas it is about twice at 100 rad/s. This trend is consistent with that observed for steady shear viscosity results.

#### 3.1.2. Film Microstructure and Micro-Texture

**Figure 5:**
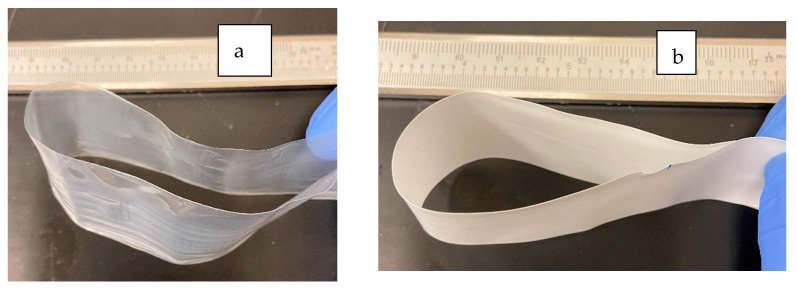

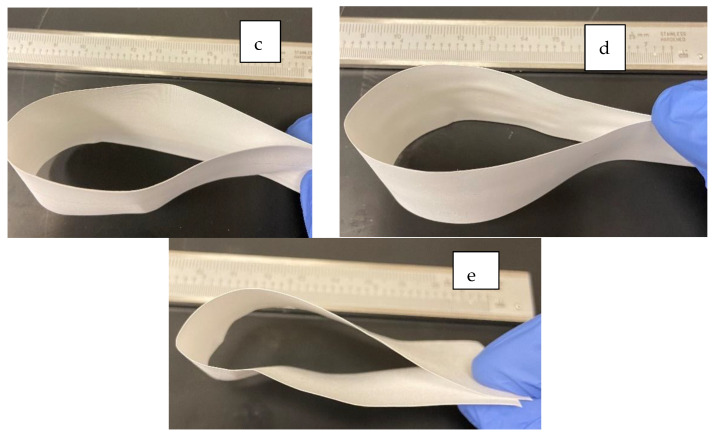
Representative pictures of folded films displaying flexibility for various BN contents: (**a**) neat LLDPE (0% BN), (**b**) 5 vol%, (**c**) 10 vol%, (**d**) 20 vol%, and (**e**) 30 vol%.

Figure 5 displays photographs of textured films produced using a range of BN contents (0 to 30 vol%) The maximum amount of BN in the LLDPE matrix for successful film extrusion was 30 vol%. Larger BN concentrations were attempted but could not be conducted due to excessive pressure drop that reached the instrument limit. This trend is consistent with the trend of increasing viscosity values presented earlier in Figure 2. Tensile strength of the neat LLDPE film was found to be 23 ± 0.4 MPa. As expected, addition of discontinuous reinforcements typically reduced ductility and tensile strength, which was measured at 20 ± 1, 19 ± 2 and 17 ± 1 MPa for 10, 20, and 30 vol% BN nanoplatelets. Additionally, as expected, tensile modulus increased from 456 ± 2.3 MPa for neat LLDPE to 455 ± 111, 599 ± 97, and 1267 ± 244 MPa for 10, 20 and 30 vol% BN. Tensile modulus for textured film was about 20 to 40% lower than non-textured films, likely due to BN platelets distributed in the texture of the film. Tensile strength of the textured films did not deteriorate due to texturing, i.e., there was no statistically significant difference between tensile strength of textured and non-textured films. Moreover, even 30 vol% BN films possessed significant flexibility as demonstrated by films in Figure 5 that show representative pictures of the films bent over without breaking even at the highest 30 vol% BN content.

**Figure 6:**
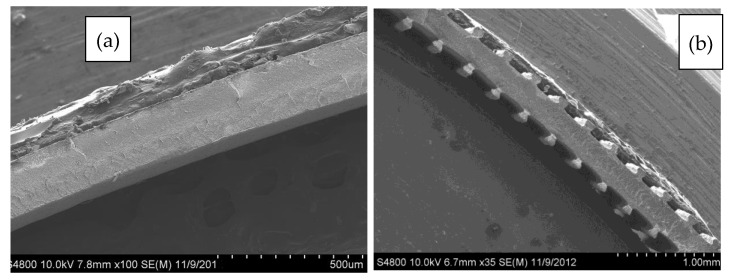
Representative SEM micrographs that showing the cross-sectional area of (**a**) non-textured, and (**b**) micro-textured BN/LLDPE films containing 30 vol% BN.

**Figure 7:**
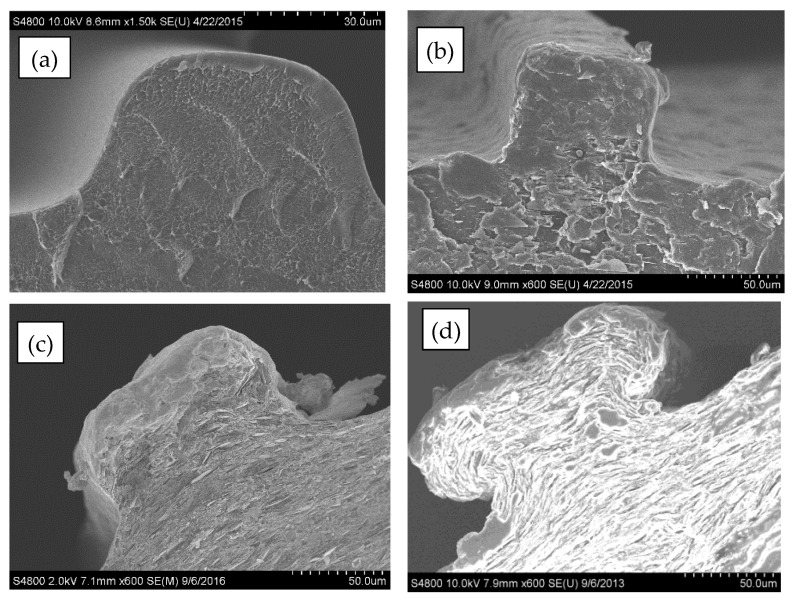
Representative SEM micrographs of cross-section of a single texture of micro-textured: (**a**) neat LLDPE, (**b**) BN5-LLDPE, (**c**) BN10-LLDPE, and (**d**) BN30-LLDPE films.

SEM micrographs of the cross-sectional area of the neat and 30 vol% BN films are displayed in Figure 6. The film micro-texture heights were measured at 70 ± 18 µm. Further, micrographs of single textures are displayed in Figure 7a–d, which show that the shape of film micro-texture is slightly different for films containing different BN contents. The shape resembled more closely the die shape as more BN was incorporated into LLDPE. This shape similarity is a result of decreased effect of polymer molecular elasticity and consistent with earlier die swell results where BN decreased molecular elasticity (reduced die swell) due to increased textural elasticity of the composite. As is also evident from Figure 7, texturing led to an extended lateral surface. This resulted in about 11% increase in area for neat LLDPE, 25% increase for 10 vol% BN, 30% for 20 vol% BN, and 45% increase in area for textured films containing 30 vol% BN. 

The importance of this increased surface area lies in the fact that convective heat dissipation rate is determined as the product U×A×ΔT, where U is overall heat transfer coefficient, A is area of heat transfer, and ΔT is the temperature difference driving the thermal transport. The term U×A consists of the film-side coefficient h_i_A_i_, and so increased area due to micro-textures directly increases A_i_, and thence the heat transport rate.

**Figure 8:**
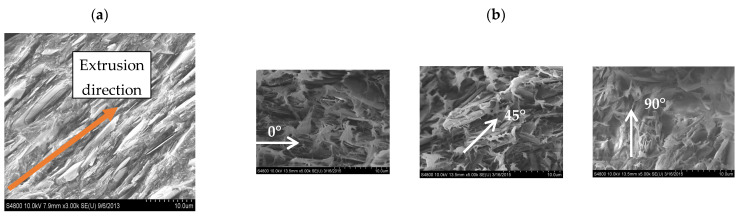
Representative SEM micrographs showing BN orientation in cross-section of BN-filled LLDPE films: (**a**) extrusion direction, and (**b**) textured microfeatures.

With regards to BN orientation, Figure 8a reveals that the platelets were aligned parallel to the main film direction due to shear and extensional stresses encountered during extrusion and film take-up. The orientation of the platelets within the fins was partially in the radial direction due to the shear stress inside the die cavity. As illustrated in SEM micrographs presented in Figure 8b, BN platelets orientation was measured inside the fins and found to be nominally 4°, 27°, and 90° at 0°, 45°, and 90°, respectively, in reference to the extrusion/flow direction shown by the arrow in Figure 8a. Thus, the orientation of microplatelets within the fins is in the circumferential direction due to the shear stress inside the die cavity.

### 3.2. Thermal Conductivity of BN/LLDPE Films

**Figure 9:**
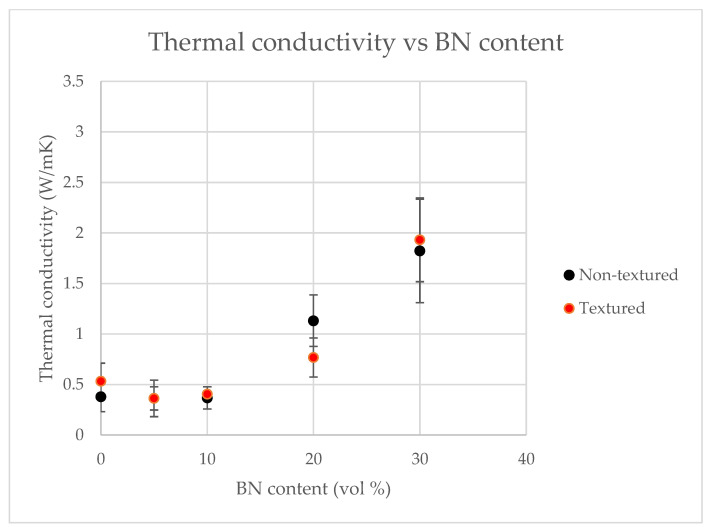
Through-thickness thermal conductivities of textured and non-textured composite LLDPE films at different BN content and Lewis–Nielsen model fitted with the data.

Through-thickness thermal conductivity of the pure polymer was measured to be 0.3 W/m·K and it increased more than five times for the composites containing 30 vol% BN, as shown in Figure 9.

**Figure 10:**
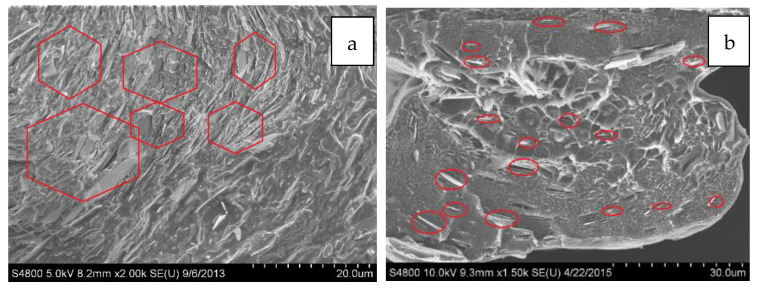
SEM image highlighting BN platelets alignment in the cross-section area of the film containing: (**a**) 30 vol% and (**b**) 5 vol%.

As expected, addition of thermally conductive BN enhances thermal conductivities for both textured and non-textured films. Figure 10 shows the BN nanoplatelet alignment in BN5 and BN30 samples. Although there are some variations in the orientation of BN nanoplatelets in the two types, no deterioration of thermal conductivity was observed for the textured films (relative to its non-textured counterpart). However, a major advantage of textured films is that texturing leads to up to 46% increase in surface area for through-thickness thermal transport, thus increasing heat transfer rate as discussed in Section 3.1.2. It is further noted that addition of BN leads to an increase in thermal conductivity (k_wall_) of wall material through which transport takes place, and thus increases the wall thermal conductance (k_wall_/Δx_wall_). Thus, combined effect of 5-fold increase in thermal conductivity and up to 50% increase in area of heat transfer will lead to significant increase in the overall convective heat transfer rates.

## 4. Conclusions

From the steady shear study of the BN-filled LLDPE, it was found that there was an expected increase in viscosity with increase in BN content. This increase in viscosity created a large pressure drop across the extrusion die, which limited the BN content to about 30 vol%. However, with increasing BN content, die swell decreased. This explains why the micro-texture of the resulting films conformed well to the shape of the die micro-pattern at high BN contents. Interestingly, this indicates that a complex-shape die may not be essential for extruding nanocomposites with micro-patterns, in contrast to that needed for neat (unfilled) polymers.

As expected, tensile modulus of 30 vol% BN-filled LLDPE was almost three times that of pure LLDPE, whereas the tensile strength showed a moderate decrease that is typically observed for particle-filled polymers. Through-thickness thermal conductivity was measured at 1.8 W/m.K for films containing 30 vol% BN, which is about a 500% enhancement as compared with the neat LLDPE. Micro-texturing did not cause any significant change in thermal conductivity but led to as much as a 45% increase in surface area. This translates to significant increase in convective heat dissipation because the convective heat transport rate scales directly with surface area. Thus, BN-filled LLDPE micro-textured films with extended surface area (50% increase) were successfully produced using a continuous film extrusion process. This study establishes a novel approach that is scalable to continuous film manufacturing for enhanced thermal transport. 

## Figures and Tables

**Table 1:** Cross model fitting parameters of BN/LLDPE blends.

	LLDPE	BN1	BN5	BN10	BN20	BN30
n	0.50	0.50	0.45	0.45	0.40	0.38
λ (s)	0.005	0.05	0.050	0.055	0.060	0.070
Ƞ_0_ (Pa.s)	467	1391	2337	3000	4600	7600

## Data Availability

The data presented in this study are available on request from the corresponding author.
